# Involvement of phosphatase and tensin homolog deleted from chromosome 10 in rodent model of neuropathic pain

**DOI:** 10.1186/s12974-015-0280-1

**Published:** 2015-03-26

**Authors:** Shi-Ying Huang, Chun-Sung Sung, Wu-Fu Chen, Chun-Hong Chen, Chien-Wei Feng, San-Nan Yang, Han-Chun Hung, Nan-Fu Chen, Pey-Ru Lin, San-Cher Chen, Hui-Min David Wang, Tian-Huei Chu, Ming-Hong Tai, Zhi-Hong Wen

**Affiliations:** Department of Marine Biotechnology and Resources, National Sun Yat-sen University, No. 70, Lienhai Road, Kaohsiung, 80424 Taiwan; Center for Neuroscience, National Sun Yat-sen University, No. 70, Lienhai Road, Kaohsiung, 80424 Taiwan; Department of Anesthesiology, Taipei Veterans General Hospital, No. 201, Section 2, Shipai Road, Taipei, 11217 Taiwan; School of Medicine, National Yang-Ming University, No. 155, Section 2, Linong Street, Taipei, 11221 Taiwan; Department of Neurosurgery, Kaohsiung Chang Gung Memorial Hospital and Chang Gung University College of Medicine, No. 123, DAPI Road, Kaohsiung, 83301 Taiwan; Department of Neurosurgery, Xiamen Chang Gung Memorial Hospital, No. 123, Xiafei Road, Fujian, 361026 China; Doctoral Degree Program in Marine Biotechnology, National Sun Yat-sen University and Academia Sinica, No. 70, Lienhai Road, Kaohsiung, 80424 Taiwan; School of Medicine, College of Medicine and Department of Pediatrics, E-DA Hospital, I-Shou University, No. 1, Yida Road, Kaohsiung, 82445 Taiwan; Division of Neurosurgery, Department of Surgery, Kaohsiung Armed Forces General Hospital, No. 2, Zhongzheng 1st Road, Kaohsiung, 80284 Taiwan; Institute of Biomedical Sciences, National Sun Yat-sen University, #70 Lienhai Road, Kaohsiung, 80424 Taiwan; Department of Fragrance and Cosmetic Science, Kaohsiung Medical University, No. 100, Shiquan 1st Road, Kaohsiung, 80708 Taiwan; Graduate Institute of Natural Products, Kaohsiung Medical University, No. 100, Shiquan 1st Road, Kaohsiung, 80708 Taiwan; Center for Stem Cell Research, Kaohsiung Medical University, No. 100, Shiquan 1st Road, Kaohsiung, 80708 Taiwan; Department of Biological Sciences, National Sun Yat-sen University, No. 70, Lienhai Road, Kaohsiung, 80424 Taiwan; Marine Biomedical Laboratory and Center for Translational Biopharmaceuticals, Department of Marine Biotechnology and Resources, National Sun Yat-sen University, No. 70, Lienhai Road, Kaohsiung, 80424 Taiwan

**Keywords:** Chronic constriction injury, Intrathecal, Astrocyte, Neuroinflammation

## Abstract

**Background:**

Many cancer research studies have extensively examined the phosphatase and tensin homolog deleted from chromosome 10 (PTEN) pathway. There are only few reports that suggest that PTEN might affect pain; however, there is still a lack of evidence to show the role of PTEN for modulating pain. Here, we report a role for PTEN in a rodent model of neuropathic pain.

**Results:**

We found that chronic constriction injury (CCI) surgery in rats could elicit downregulation of spinal PTEN as well as upregulation of phosphorylated PTEN (phospho-PTEN) and phosphorylated mammalian target of rapamycin (phospho-mTOR). After examining such changes in endogenous PTEN in neuropathic rats, we explored the effects of modulating the spinal PTEN pathway on nociceptive behaviors. The normal rats exhibited mechanical allodynia after intrathecal (i.t.) injection of adenovirus-mediated PTEN antisense oligonucleotide (Ad-antisense PTEN). These data indicate the importance of downregulation of spinal PTEN for nociception. Moreover, upregulation of spinal PTEN by i.t. adenovirus-mediated PTEN (Ad-PTEN) significantly prevented CCI-induced development of nociceptive sensitization, thermal hyperalgesia, mechanical allodynia, cold allodynia, and weight-bearing deficits in neuropathic rats. Furthermore, upregulation of spinal PTEN by i.t. Ad-PTEN significantly attenuated CCI-induced microglia and astrocyte activation, upregulation of tumor necrosis factor-α (TNF-α) and phospho-mTOR, and downregulation of PTEN in neuropathic rats 14 days post injury.

**Conclusions:**

These findings demonstrate that PTEN plays a key, beneficial role in a rodent model of neuropathic pain.

## Background

Pain affects 1.5 billion people globally, including 116 million people in the USA and 164 million people in Europe and Israel combined [1]. The 2009 global pain market was estimated to be over US $50 billion [2]. Previous studies indicated that chronic pain occurs in about 20% of the general population [3,4], and the prevalence of neuropathic pain is 6.9% [4]. In a review of 174 trials published, Finnerup *et al*. [5] reported that there are no drug treatments available that can relieve all neuropathic pain conditions. Moreover, the detailed mechanisms underlying neuropathic pain still remain unclear.

The phosphatase and tensin homolog deleted from chromosome 10 (PTEN) is a tumor suppressor of phosphatased activity [6]. PTEN has been studied extensively through cancer research [7,8]; however, there are only few reports that suggest that PTEN might affect pain [9]. Goebbels *et al*. have demonstrated that by targeted disruption of *Pten* in Schwann cells that causes focal hypermyelination in the PNS and is associated with progressive peripheral neuropathy in mice [9]. However, there is still a lack of evidence to show the role of PTEN for modulating pain. PTEN is considered an upstream inhibitory mediator of mammalian target of rapamycin (mTOR) [10-12]. Several studies have demonstrated that inhibition of the spinal mTOR pathway can attenuate nociception in neuropathic pain [13,14]. We suspect that PTEN upregulation may have a therapeutic effect on neuropathic pain.

Here, we use a rat model of chronic constriction injury (CCI)-induced neuropathic pain combined with an intrathecal (i.t.) delivery system to determine whether central PTEN plays a role in neuropathic pain. We examined changes in endogenous spinal PTEN in neuropathic rats using our adenovirus-mediated target gene overexpression system. This system has been shown to be stable and effective in rodent models, especially adenovirus-mediated PTEN (Ad-PTEN) in *in vitro* mouse models [15] and in both *ex vivo* [16] and *in vivo* [17] rat models. Additionally, we examined whether modulating the spinal PTEN pathway affected nociceptive behaviors that were measured reliably with behavioral pain assays [18,19]. Spinal neuroinflammation may accelerate central sensitization and promote the development and maintenance of neuropathic pain [20,21]. Furthermore, spinal neuroinflammation in CCI is characterized by microglial and astrocytic activation and increased expression of the proinflammatory mediator tumor necrosis factor-α (TNF-α) [19,22]. Many studies have further demonstrated that inhibiting microglial and astrocytic activation can have analgesic effects [19,23-25]. Similarly, TNF-α reportedly plays key roles in neuropathic pain [26,27], whereas, inhibition of spinal TNF-α inhibits neuropathic pain behavior [28]. In the present study, we examine whether PTEN affects spinal microglial and astrocytic activation and upregulation of TNF-α accompanied the nociceptive behaviors in CCI rats. In addition, we examine whether PTEN affects downstream signaling of mTOR at the spinal level and in the neuropathic state.

## Methods

### Animals

We housed the male Wistar rats (260 to 285 g) for free access to food and water in a temperature-controlled (22°C ± 1°C) and light-cycle-controlled (12-h light/12-h dark) room. After the approval by the National Sun Yat-sen University and Use Committee, we conformed to the Guiding Principles in the Care and Use of Animals of the American Physiology Society to use rats throughout the experiments. For surgery and drug injections, all rats were anesthetized under isoflurane inhalation (2%). Then, for preventing infection during the surgery, all rats received intramuscularly postoperative injection of veterin (cefazolin; 0.17 g/kg). Our every effort in experimental design and execution was on the purpose of minimizing the suffering and number of rats we used.

### Induction of peripheral mononeuropathy (CCI)

As described by Bennett and Xie [29] and our previous studies [19,23], we performed the surgery of CCI to the right sciatic nerve of rats, to expose the right sciatic nerve of rats (at mid-thigh level), to dissect a 5-mm-long nerve segment of the sciatic nerve, to place four loose ligatures (4 to 0 chromic gut) around the sciatic nerve (with 1-mm intervals), and then to suture muscle and skin incision layer by layer. For the sham-operated rats, the surgery was performed only to expose the right sciatic nerve but without ligation.

### Implantation of i.t. catheters

Using the method described in Yaksh and Rudy [30] and our previous studies [19,23], we inserted i.t. catheter (PE5 tubes: 9-cm long, 0.008-in. inner diameter, 0.014-in. outer diameter; Spectranetics, Colorado Springs, CO, USA) to the lumbar enlargement of the spinal cord via the atlanto-occipital membrane at the base of the rat’s skull down, and then externalized and fixed one end of the i.t. catheter to the cranial aspect of the rat’s head for spinal drug administration. Because the dead volume of i.t. catheter was 3.5 μL, to ensure complete drug delivery, an i.t. artificial cerebrospinal fluid (aCSF) flush (10 μL) followed all i.t. injections. The composition of aCSF is as follows: 151.1 mM Na^+^, 2.6 mM K^+^, 1.3 mM Ca^2+^ , 0.9 mM Mg^2+^, 122.7 mM Cl^−^, 21.0 mM HCO_3_^−^, 2.5 mM HPO_4_^2−^, and 3.5 mM dextrose and bubbled with 5% CO_2_ in 95% O_2_ for adjusting the final pH to 7.3. Five days after implantation of i.t. catheters, we excluded rats with i.t. catheter that had the fresh blood in the CSF or exhibit of gross neurological injury from the following experiments. According to the method described in other previous study [31] and our previous studies [19,23], we evaluated the locomotor function of rats using the Basso, Beattie, and Bresnahan (BBB) locomotor scale [32].

### Preparation of adenovirus vectors

Using the method described in our previous reports [15-17], we prepared E1- and E3-defective recombinant adenovirus vectors encoding green fluorescent protein (Ad-GFP), PTEN antisense oligonucleotide (Ad-antisense PTEN), or human PTEN cDNA (Ad-PTEN). These adenovirus solutions were tittered with a plaque-forming assay, aliquoted, and then stored at −80°C before their use.

### Nociceptive behavioral testing

(i.) Thermal hyperalgesia test: After we placed each rat into each compartment of clear plastic cages onto an elevated glass platform, we then used an IITC analgesiometer (IITC Inc., Woodland Hills, CA, USA) to test thermal hyperalgesia as described previously by Hargreaves *et al*. [33] and our previous study [18,34] to position a radiant heat source with low-intensity heat (active intensity = 25) onto the middle of the plantar surface of the rat, with a cutoff time of 30 s, to measure the paw withdrawal latency (PWL; in seconds) of the rat until the rat showed a positive sign of pain behavior (licking or withdrawal).

(ii.) Mechanical allodynia test: After we set each rat into each compartment of clear plastic cages onto an elevated metal mesh floor for easy access to the rats’ paws, we measured hindpaw withdrawal threshold (PWT; in g) to assess mechanical allodynia using calibrated von Frey filaments (Stoelting, Wood Dale, IL, USA). Then, we apply a series of von Frey filaments of logarithmically incremental stiffness to the midplantar region of the rat hindpaw by Chaplan’s ‘up-down’ method to determine the closest filament to the threshold of pain response (licking or withdrawal) of the rat as described previously by Chaplan *et al*. [35] and our previous studies [18,19].

(iii.) Cold allodynia test: As described previously by our previous study [18], after we placed each rat in the individual plastic compartments on an elevated metal mesh floor, cold allodynia response (acetone response score; in point) of the rat was monitored during 1 min following acetone stimulus (25 μl) onto the center of the plantar surface of rats’ hindpaw. Modified from 4-point scale of previous studies [36,37], acetone response scores of the paw were graded according to a 6-point scale: 0, repeated flicking with persistent licking in 2 s following acetone stimulus; 1, prolonged withdrawal or repeated flicking in 2 s following acetone stimulus; 2, quick and more violent withdrawal, flick, or stamp in 2 s following acetone stimulus; 3, quick withdrawal, flick, or stamp in 2 s following acetone stimulus; 4, withdrawal, flick, or stamp more than 2 s following acetone stimulus; and 5, no response. Then, to sum the four individual scores for obtaining acetone response score of the paw of each rat. The minimum possible total score of the paw could be 0 point (the rat exhibits repeated flicking and licking of paws on each of the four trials), and the maximum possible total score of the paw could be 20 points (the rat exhibits no response to any of the four trials).

(iv.) Weight-bearing test: To let the rat place its hindpaws onto the two force transducers of the incapacitance tester (Singa Technology Corporation, Taoyuan, Taiwan, Republic of China) for measuring the hindpaw weight-bearing deficits (change in hindpaw weight distribution; in g) as described in our previous study [18,38]. Under normal conditions, the naïve rat distributes weight equally between both hind limbs, but after inducing inflammation of one hind limb (like CCI), the rat redistributes weight for lowering weight-bearing of the affected limb [39]. Change in hindpaw weight distribution of the rat was expressed as a difference by subtracting the affected limb (the right hind limb; the ipsilateral side) from the normal limb (the left hind limb; the contralateral side) measured at the same time.

### Spinal immunohistofluorescence analysis

For reducing variations in immunohistochemical procedures, we mounted the lumbar spinal tissues from the different groups of rats into the same OCT block, sectioned these spinal tissues together using a cryostat at −30°C (HM550; Microm, Waldorf, Germany) and performed the following spinal immunohistofluorescence analysis, with a modified method described in Sung *et al*. [40] and our previous studies [18,23]. The spinal sections (10 μm) were incubated with primary antibody, anti-phosphorylated PTEN (anti-phospho-PTEN; Ser380) (1:200 dilution, cat. 9551; Cell Signaling Technology Inc., Beverly, MA, USA; polyclonal rabbit antibody), anti-PTEN (1:200 dilution, cat. 10005059; Cayman Chemical, Ann Arbor, MI, USA; polyclonal rabbit antibody), anti-phosphorylated mTOR (anti-phospho-mTOR; Ser2448) (1:200 dilution, cat. 2976; Cell Signaling Technology Inc., Beverly, MA, USA; polyclonal rabbit antibody), anti-OX-42 (CD11b, microglial marker, 1:200 dilution, cat. CBL1512; EMD Millipore, Temecula, CA, USA; monoclonal mouse antibody), anti-glial fibrillary acidic protein (GFAP) (astrocytic marker, 1:200 dilution, cat. MAB3402; EMD Millipore, Temecula, CA, USA; monoclonal mouse antibody), or anti-TNF-α (1:200 dilution, cat. ARC3012; Life Technologies Corporation, Grand Island, NY, USA) overnight at 4°C. This was then followed by Alexa Fluor 488-labeled chicken anti-mouse IgG antibody (1:400 dilution, cat. A-21200; Molecular Probes, Eugene, OR, USA; green fluorescence) or DyLight 549-conjugated donkey anti-rabbit IgG antibody (1:400 dilution, cat. 711-506-152; Jackson ImmunoResearch Laboratories Inc., West Grove, PA, USA; red fluorescence) for 40 min at room temperature. For immunostaining analysis, we use a Leica DM-6000 CS fluorescence microscope (Leica Instruments Inc., Wetzlar, Germany) for examination of these stained spinal sections, and then use a SPOT Xplorer Digital camera (Diagnostic Instruments, Inc., Sterling Heights, MI, USA) for photographing all immunofluorescence images of phospho-PTEN, PTEN, phospho-mTOR, OX-42, GFAP, and TNF-α, respectively. We measured the pixel values of the immunoreactive-positive area (using three sections per rat) by Image J software (National Institutes of Health, Bethesda, MD, USA). Spinal neurons located in the superficial laminae, laminae I to III, respond to nociceptive stimuli and directly participate in the transmission of nociception to the brain [41], and thereby the superficial laminae play a more important role in neuropathic pain than the deep laminae. Therefore, in accordance to the method used for neuropathic rodents [41-44], we focused on quantifying the immunoreactivity of the targeted protein in the superficial laminae of the spinal cord. The immunofluorescence data were expressed as a percentage change compared to sham-operated or sham-operated plus i.t. vehicle group, which were considered to be 100%. For double-immunofluorescent staining of PTEN and neuronal marker, the spinal sections were incubated with a mixture of anti-PTEN (1:200 dilution) and anti-Neuronal Nuclei (NeuN) (neuronal-specific nuclear protein; neuronal marker, 1:500, Alexa Fluor 488 conjugated antibody, cat. MAB377X, EMD Millipore, Temecula, CA, USA; monoclonal mouse antibody) antibodies overnight at 4°C, and then followed by DyLight 549-conjugated anti-rabbit IgG antibody (1:400 dilution) for 40 min at room temperature. On the other hand, for double-immunofluorescent staining of PTEN and microglial marker or astrocytic marker, the spinal sections were incubated with a mixture of anti-PTEN (1:200 dilution) and OX-42 (1:200 dilution) or GFAP (1:200 dilution) antibodies overnight at 4°C, and then followed by a mixture of Alexa Fluor 488-conjugated anti-mouse IgG antibody (1:400 dilution) and DyLight 549-conjugated anti-rabbit IgG antibody (1:400 dilution) for 40 min at room temperature. For double-immunofluorescent staining of anti-TNF-α or phospho-mTOR and astrocytic marker, the spinal sections were incubated with a mixture of anti-TNF-α (1:200 dilution) or anti-phospho-mTOR (1:200 dilution) and GFAP (1:200 dilution) antibodies overnight at 4°C, and then followed by a mixture of DyLight 549-conjugated anti-rabbit IgG antibody (1:400 dilution) and Alexa Fluor 633-conjugated goat anti-mouse IgG antibody (1:400 dilution, cat. A21052; Life Technologies Corporation) for 40 min at room temperature. The double-immunostaining images were examined and acquired with Leica TCS SP5 II confocal microscope (Leica Instruments Inc., Wetzlar, Germany). We set the color of TNF-α or phospho-mTOR for 549-nm excitation line as pseudo red and set the color of GFAP for 633 nm excitation line as pseudo green. When co-localization of two proteins occurred, the merge of above two colors, pseudo red (TNF-α or phospho-mTOR) and pseudo green (GFAP), yielded yellow color.

### Western blot analysis

Following our published method [22,34,45], we performed Western blotting analysis on the ipsilateral side of the lumbar spinal dorsal samples from the rats. For Western blotting analysis, we collected and washed spinal dorsal samples from rats with ice-cold PBS, and then used a Polytron homogenizer (5 cycles of 10 s at 3,000 rpm) to homogenize in ice-cold lysis buffer (1 μg/ml aprotinin, 50 mM Tris, 150 mM NaCl, 100 μg/ml phenylmethylsulfonyl fluoride, 1% Triton X-100, pH 7.5). After centrifuging at 20,000 × *g* for 60 min at 4°C, we retained this supernatant for Western blot analysis of phospho-PTEN and PTEN. Modified from the method of Lowry *et al*. [46], we determined the protein concentrations of the supernatant with the DC protein assay kit (Bio-Rad, Hercules, CA, USA). We added an equal volume of sample buffer (2% 2-mercaptoethanol, 10% glycerol, 0.1% bromophenol blue, 50 mM Tris–HCl, pH 7.2, and 2% sodium dodecyl sulfate (SDS)) to the supernatant. Then, we electrophoresed the proteins of supernatant through a tricine SDS-polyacrylamide gel at 150 V for 90 min and transferred the proteins within the gel to a polyvinylidene difluoride membrane (PVDF membrane; Immobilon-P, Millipore, 0.45-μM pore size) in transfer buffer (380 mM glycine, 1% SDS, 50 mM Tris–HCl, 20% methanol) at 125 mA overnight at 4°C. With blocking PVDF membrane for 1 h at room temperature using 5% non-fat dry milk in Tris-buffered saline (TTBS; 137 mM NaCl, 0.1% Tween 20, 20 mM Tris–HCl, pH 7.4), we then incubated the PVDF membrane with antibodies against phospho-PTEN (Ser380; 1:1,000 dilution) or PTEN (1:1,000 dilution) proteins for 180 min at room temperature. The antibodies recognized immunoreactive bands of phospho-PTEN (approximately 54 kDa) and PTEN (approximately 47 kDa) protein, which were visualized with enhanced chemiluminescence (ECL kit; Millipore) and photographed using the UVP BioChemi imaging system (UVP LLC, Upland, CA, USA), respectively. Finally, we performed the relative densitometric quantification of the immunoreactive bands of phospho-PTEN and PTEN protein with LabWorks 4.0 software (UVP LLC, Upland, CA, USA), and relative variations between the bands of the sham-operated or sham-operated plus i.t. vehicle group and the other groups were calculated using the same image. In addition, we reprobed the PVDF membranes using an anti-β-actin antibody (1:2,500 dilution; catalog no. A5441; Sigma Co., Ltd., St Louis, MO, USA; monoclonal mouse antibody), which was the loading control.

### Data and statistical analysis

We showed all following data as means ± standard error on the mean (SEM). For statistical analysis, we calculated differences between groups of rats by a one-way analysis of variance (ANOVA), used the Student-Newman-Keuls *post hoc* test, and then defined the criterion for statistical significance as *P* < 0.05.

## Results

### The time course of changes of endogenous spinal PTEN in neuropathic rats

**Figure 1:**
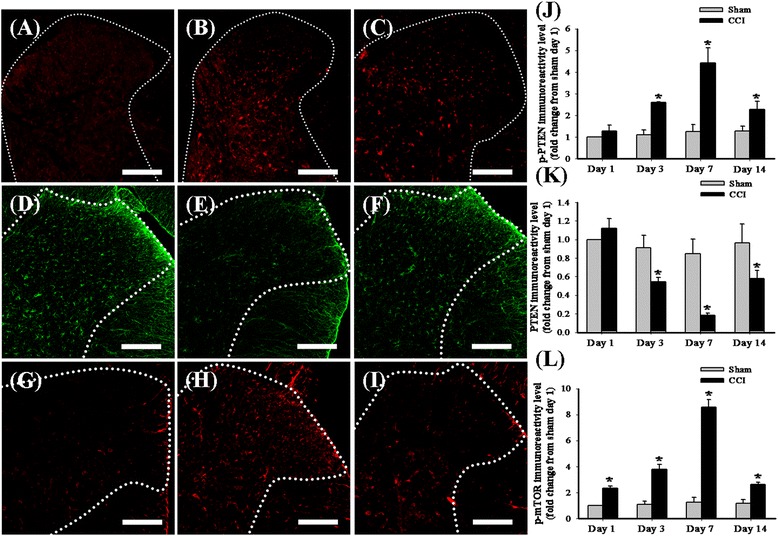
**The time course effects of CCI on spinal phospho-PTEN, PTEN, and phospho-mTOR.** Spinal cord sections (10 μm) were from the sham-operated **(A, D, G)**, 7 days after CCI **(B, E, H)**, and 14 days after CCI **(C, F, I)** groups. Immunostaining images show cells labeled with phospho-PTEN (red, **(A-C)**), PTEN (green, **(D-F)**), and phospho-mTOR (red, **(G-I)**) in the spinal cord. Quantification of phospho-PTEN **(J)**, PTEN **(K)**, and phospho-mTOR **(L)** immunoreactivity in the ipsilateral dorsal horn of the lumbar spinal gray matter 1, 3, 7, and 14 days after CCI as compared with sham-operated rats. Each bar in panels **(J-L)** represents the mean ± SEM; six rats per group. CCI significantly upregulated spinal immunoreactivity of phospho-PTEN and phospho-mTOR and downregulated spinal PTEN immunoreactivity. CCI, chronic constriction injury; p-mTOR, phosphorylated mammalian target of rapamycin; PTEN, phosphatase and tensin homolog deleted from chromosome 10; p-PTEN, phosphorylated PTEN. Scale bars: 200 μm for all images **(A-I)**. *P < 0.05 compared with the sham-operated group.

**Figure 2:**
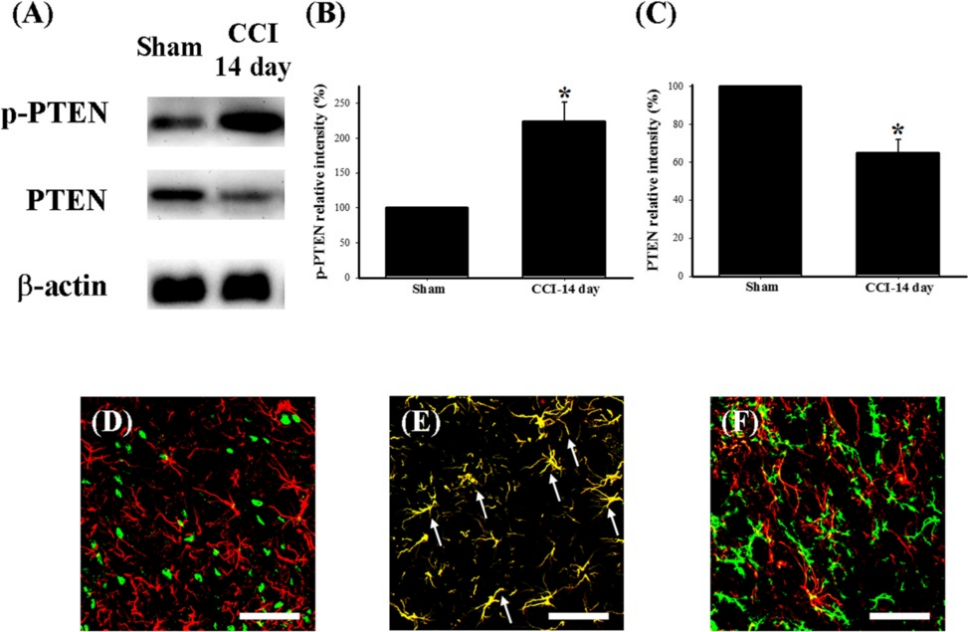
**Changes in cellular specificity of endogenous PTEN in the dorsal horn of the spinal cord. (A)** Western blots for phospho-PTEN, PTEN, and β-actin proteins from sham-operated and 14 days after CCI groups; **(B)** relative density of immunoblot of phospho-PTEN; **(C)** relative density of immunoblot of PTEN. Relative band intensities of phospho-PTEN and PTEN were quantified by densitometry and indicated as the percent change relative to that for the sham-operated group (100%). Western blotting of spinal dorsal horn tissue homogenate revealed that phospho-PTEN and PTEN protein expression was detectable in the sham-operated group, but CCI evoked upregulation of phospho-PTEN protein and downregulation of PTEN protein 14 days after CCI. Western blotting of β-actin was performed to verify the equivalent amounts of protein were loaded in each lane. Confocal double-immunofluorescent staining of PTEN (red) with NeuN (neuronal-specific marker; **(D)**, green), GFAP (astrocyte specific marker; **(E)**, green) OX-42 (microglial specific marker; **(F)**, green) in the dorsal horn region of the lumbar spinal cord of sham-operated group. The merged images of **(D)**, **(E)**, and **(F)** (yellow; white arrow) indicate co-localization of PTEN with NeuN, GFAP, and OX-42 immunoreactive cells in the spinal cord, respectively. The confocal results showed that spinal PTEN was primarily co-localized with astrocytes. Scale bars are 50 μm for all images **(D-F)**. Each bar in **(B)** and **(C)** represents the mean ± SEM with three rats per group. CCI-14 day: 14 days after CCI. *P < 0.05 compared with sham-operated group. CCI, chronic constriction injury; PTEN, phosphatase and tensin homolog deleted from chromosome 10; p-PTEN, phosphorylated PTEN.

**Figure 3:**
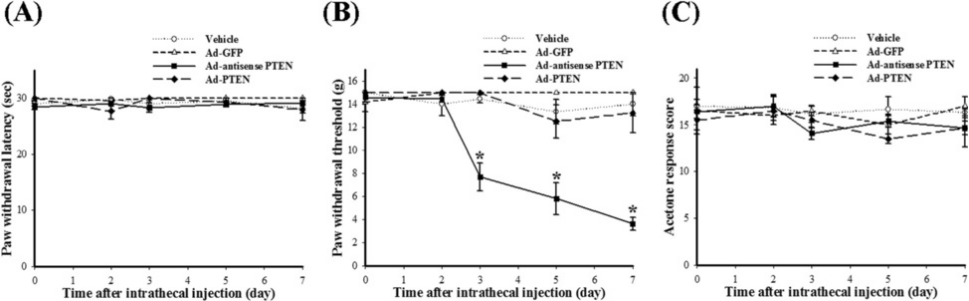
**Time course of the effects of i.t. Ad-antisense PTEN on nociceptive behaviors in normal rats.** The naïve rats exhibited mechanical allodynia **(B)** but not thermal hyperalgesia **(A)** and cold allodynia **(C)** from 3 to 7 days after i.t. injection of Ad-antisense PTEN. These data indicate the importance of downregulation of spinal PTEN for nociception. Each point represents the mean ± SEM with six rats per group. *P < 0.05 compared with vehicle group. Ad-GFP, adenovirus-mediated green fluorescent protein; Ad-PTEN, adenovirus-mediated phosphatase and tensin homolog deleted from chromosome 10; Ad-antisense PTEN, adenovirus-mediated PTEN antisense oligonucleotide.

To explore any potential changes in the spinal PTEN pathway in neuropathic rats, we prepared the lumbar spinal tissues from the following eight groups of rats: (1) 1 day after sham operation, (2) 3 days after sham operation, (3) 7 days after sham operation, (4) 14 days after sham operation, (5) 1 day after CCI, (6) 3 days after CCI, (7) 7 days after CCI, and (8) 14 days after CCI. We performed a spinal immunohistofluorescence analysis using three antibodies against phospho-PTEN, PTEN, and phospho-mTOR to evaluate the expression of the PTEN pathway in the ipsilateral side of the lumbar spinal dorsal gray matter in neuropathic rats. Compared with the sham-operated group (Figure 1A,D), immunoreactivity increased for phospho-PTEN but decreased for PTEN, 7 (Figure 1B,E) and 14 days (Figure 1C,F) after CCI. Similar to the findings in a previous study [47], phospho-mTOR immunoreactivity increased 7 (Figure 1H) and 14 days (Figure 1I) after CCI, as compared with that of the sham-operated group (Figure 1G). Quantification of the immunoreactivity results further confirmed phospho-PTEN (Figure 1J) and phospho-mTOR (Figure 1L) upregulation, as well as PTEN downregulation (Figure 1K), 3, 7, and 14 days after CCI. Further, we used Western blot analysis to verify changes in endogenous PTEN in the dorsal horn of the lumbar spinal cord (Figure 2A). The Western blot analysis revealed upregulation of phospho-PTEN (Figure 2B) and downregulation of PTEN (Figure 2C) 14 days after CCI surgery. To examine the cellular specificity of endogenous PTEN in the dorsal horn of the lumbar spinal cord, we prepared lumbar spinal tissues from the sham-operated group for double-immunofluorescent staining. Confocal double-immunostaining images of the lumbar spinal dorsal gray matter of sham-operated rats further confirmed that most PTEN signals were more often co-localized with GFAP-positive cells (astrocytes, Figure 2E) than NeuN-positive (neuronal cells; Figure 2D) or OX-42-positive cells (microglial cells, Figure 2F).

### The effect of i.t. Ad-antisense PTEN on nociceptive responses

To explore the effects of modulating spinal PTEN pathway on nociceptive behaviors in normal rats, we prepared the following five groups of rats: (1) naïve, (2) i.t. vehicle, (3) i.t. Ad-GFP, (4) i.t. Ad-antisense PTEN, and (5) i.t. Ad-PTEN. Five days after implantation of the i.t. catheters, the rats received i.t. injection of vehicle, Ad-GFP, Ad-antisense PTEN, or Ad-PTEN. No significant differences were observed between the naïve group (data not shown) and the i.t. vehicle group for PWL, PWT, or acetone response scores. Compared with i.t. vehicle group, i.t. Ad-GFP did not significantly affect PWL (Figure 3A), PWT (Figure 3B), and acetone response score (Figure 3C) of rats. After i.t. injection of Ad-antisense PTEN, the rats exhibited mechanical allodynia (Figure 3B) but not thermal hyperalgesia (Figure 3A) and cold allodynia (Figure 3C) from 3 to 7 days compared with that of i.t. Ad-GFP group. Compared with i.t. Ad-GFP group, i.t. Ad-PTEN did not significantly affect PWL (Figure 3A), PWT (Figure 3B), and acetone response score (Figure 3C) of rats. In addition, in the present study, i.t. Ad-GFP, Ad-antisense PTEN, or Ad-PTEN-treated rats did not exhibit any obvious abnormal external behavior (including locomotor function).

### The inhibitory effects of Ad-PTEN on nociception in neuropathic rats

**Figure 4:**
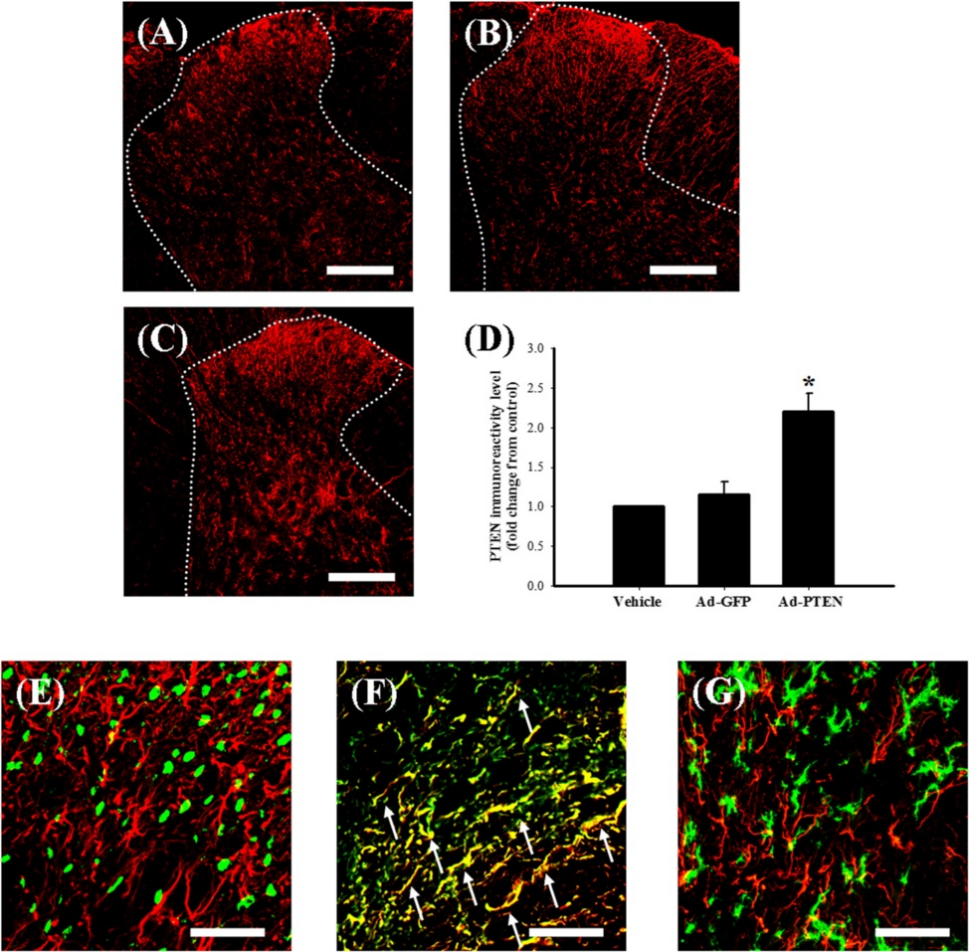
**The effects of i.t. injection of Ad-PTEN on PTEN in the dorsal lumbar spinal cord.** Fourteen days after i.t.; spinal cord sections (10 μm) are from i.t. injection of vehicle **(A)**, Ad-GFP **(B)**, and Ad-PTEN **(C)** groups. Immunostaining images of the spinal cord show cells labeled with PTEN (red). Quantification of PTEN **(D)** immunoreactivity in the ipsilateral dorsal horn of the lumbar spinal gray matter compared with vehicle group. Injection (i.t.) of Ad-PTEN significantly upregulated spinal PTEN immunoreactivity. Each bar in **(D)** represents the mean ± SEM with six rats per group. Ad-GFP, adenovirus-mediated green fluorescent protein; Ad-PTEN, adenovirus-mediated phosphatase and tensin homolog deleted from chromosome 10. Confocal double-immunofluorescent staining of PTEN (red) with NeuN (neuronal-specific marker; **(E)**, green), GFAP (astrocyte specific marker; **(F)**, green), and OX-42 (microglial specific marker; **(G)**, green) in the dorsal horn region of the lumbar spinal cord of the i.t. Ad-PTEN group. The merged images of **(E), (F)**, and **(G)** (yellow; white arrow) indicate co-localization of PTEN with NeuN, GFAP, and OX-42 immunoreactive cells in the spinal cord, respectively. The confocal results show that spinal PTEN was primarily co-localized with astrocytes in i.t. Ad-PTEN group. Scale bars are 200 μm for panels A-C and 50 μm for panels E-G. *P < 0.05 compared with the vehicle group.

To verify that Ad-PTEN has the ability to upregulate production of spinal PTEN, we prepared the following three groups of rats: (1) naïve plus i.t. vehicle, (2) naïve plus i.t. Ad-GFP, and (3) naïve plus i.t. Ad-PTEN. We observed no significant difference in spinal PTEN expression between naïve rats plus i.t. vehicle (Figure 4A) and rats examined 14 days after i.t. injection of Ad-GFP (Figure 4B), whereas PTEN immunoreactivity increased 14 days after i.t. Ad-PTEN (Figure 4C). Quantification of the immunoreactivity results (Figure 4D) further confirmed that PTEN upregulation in the naïve plus i.t. Ad-PTEN group 14 days after i.t. injection. Confocal double-immunostaining images of the lumbar spinal dorsal gray matter further confirmed most PTEN signals were more often co-localized with GFAP-positive cells (astrocytes; Figure 4F) than NeuN-positive (neuronal cells; Figure 4E) or OX-42-positive cells (microglial cells; Figure 4G) in the naïve rats administered i.t. Ad-PTEN.

**Figure 5:**
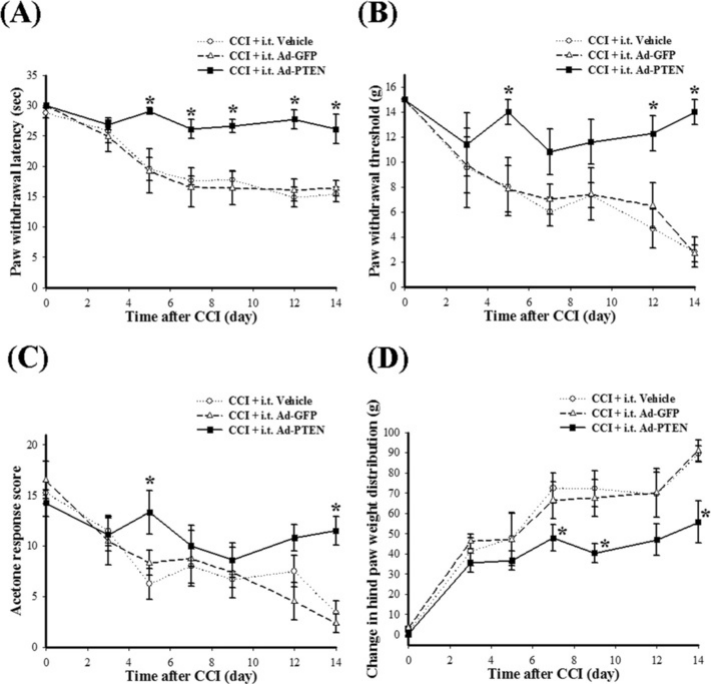
**The time course of i.t. Ad-PTEN effects on nociceptive behaviors in CCI rats.** Ad-PTEN (i.t) prevented CCI-induced development of pain behaviors with the ipsilateral hindpaw in neuropathic rats up to 14 days post injury, including thermal hyperalgesia **(A)**, mechanical allodynia **(B)**, cold allodynia **(C)**, and weight-bearing deficits **(D)**. Each point represents the mean ± SEM with six rats per group. *P < 0.05 compared with CCI plus i.t. vehicle group. Ad-GFP, adenovirus-mediated green fluorescent protein; Ad-PTEN, adenovirus-mediated phosphatase and tensin homolog deleted from chromosome 10; CCI, chronic constriction injury; i.t., intrathecal.

**Figure 6:**
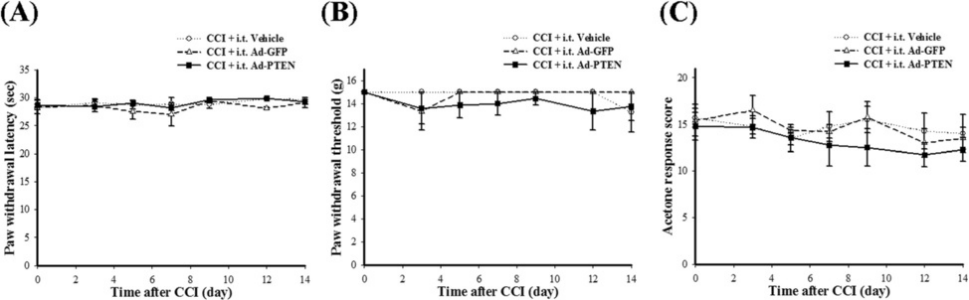
**The effects of i.t. Ad-PTEN on nociceptive behaviors with the contralateral hindpaw in CCI rats.** I.t. Ad-PTEN did not significantly affect the responses to thermal hyperalgesia **(A)**, mechanical allodynia **(B)**, or cold allodynia **(C)** tests. Each point represents the mean ± SEM with six rats per group. Ad-GFP, adenovirus-mediated green fluorescent protein; Ad-PTEN, adenovirus-mediated phosphatase and tensin homolog deleted from chromosome 10; CCI, chronic constriction injury; i.t., intrathecal.

To examine whether spinal PTEN upregulation induced with i.t. Ad-PTEN affected neuropathic pain behaviors, we prepared the following three groups of rats: (1) CCI plus i.t. vehicle, (2) CCI plus i.t. Ad-GFP, and (3) CCI plus i.t. Ad-PTEN. Five days after the i.t. catheters were implanted, the rats underwent CCI surgery, and the vehicle, Ad-GFP, or Ad-PTEN was administered i.t. immediately afterwards. Similar to previous findings [48], CCI promoted the development and maintenance of nociceptive behaviors in rats. Compared with the vehicle group, i.t. Ad-GFP did not significantly affect PWL (Figure 5A), PWT (Figure 5B), acetone response score (Figure 5C), and weight-bearing deficits (Figure 5D) for the ipsilateral hindpaw of CCI rats. Compared with i.t. Ad-GFP group, i.t. Ad-PTEN significantly attenuated CCI-induced thermal hyperalgesia (Figure 5A), mechanical allodynia (Figure 5B), cold allodynia (Figure 5C), and weight-bearing deficits (Figure 5D) up to 14 days post injury. In contrast, similar to previous studies [49,50], the contralateral hindpaw in CCI rats did not show pain-related behavior (Figure 6). Compared with i.t. vehicle group, neither i.t. Ad-GFP nor Ad-PTEN significantly affected PWL (Figure 6A), PWT (Figure 6B), or acetone response scores (Figure 6C) for the contralateral hindpaw of CCI rats. In addition, i.t. Ad-GFP- and Ad-PTEN-treated CCI rats failed to show obvious abnormal behaviors (including locomotor function), and the naïve-plus-i.t. Ad-PTEN rats exhibited normal locomotor function. We then focused on 14 days post injury to determine whether the modulation of spinal neuroinflammatory processes is involved in the maintenance of the antinociceptive effects of the upregulation of spinal PTEN by i.t. Ad-PTEN.

### The effect of Ad-PTEN on spinal neuroinflammation in neuropathic rats

**Figure 7:**
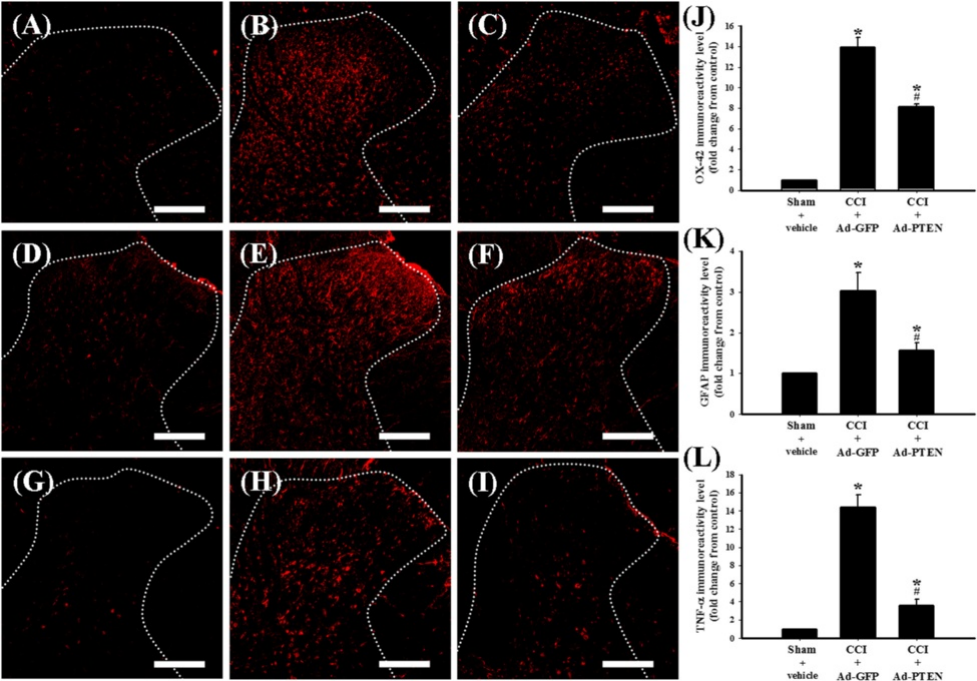
**The effects of i.t. Ad-PTEN on CCI-induced microglial and astrocytic activation and upregulation of TNF-α.** Spinal cord sections (10 μm) obtained 14 days post-surgery from sham-operated plus i.t. vehicle **(A, D, G)**, CCI plus i.t. Ad-GFP **(B, E, H)**, and CCI plus i.t. Ad-PTEN **(C, F, I)** groups. Immunostaining images show cells labeled with OX-42 (red; (A-C)) and GFAP (red; **(D-F)**), TNF-α (red; **(G-I)**) in the spinal cord. Quantification of OX-42 **(J)** and GFAP **(K)**, and TNF-α **(L)** immunoreactivity in the ipsilateral dorsal horn of the lumbar spinal gray matter. Ad-GFP, adenovirus-mediated green fluorescent protein; Ad-PTEN, adenovirus-mediated phosphatase and tensin homolog deleted from chromosome 10; CCI, chronic constriction injury; GFAP, glial fibrillary acidic protein; TNF, tumor necrosis factor. Each bar in **(J-L)** represents the mean ± SEM with six rats per group. Ad-PTEN (i.t.) significantly inhibited CCI-induced upregulation of spinal OX-42, GFAP, and TNF-α immunoreactivity. Scale bars: 200 μm for all images **(A-I)**. *P < 0.05 compared with sham-operated plus i.t. vehicle group; ^#^ *P* < 0.05 compared with CCI plus i.t. Ad-GFP.

For spinal immunohistofluorescence assay, we collected spinal tissue on day 14 post injury from the following three groups of rats: (1) sham-operated plus i.t. vehicle, (2) CCI plus i.t. Ad-GFP, and (3) CCI plus i.t. Ad-PTEN. OX-42-, GFAP-, and TNF-α-immunoreactive cells were scattered throughout the ipsilateral dorsal horn of the lumbar spinal gray matter of sham-operated plus i.t. vehicle (Figure 7A,D,G), CCI plus i.t. Ad-GFP (Figure 7B,E,H), and CCI plus i.t. Ad-PTEN (Figure 7C,F,I) groups. Similarly, and as previously reported,[19,22] the immunoreactivity of OX-42 (Figure 7B), GFAP (Figure 7E), and TNF-α (Figure 7H) of CCI plus i.t. Ad-GFP group were upregulated on day 14 post injury when compared with the sham-operated plus i.t. vehicle group. CCI-induced upregulation of OX-42 (Figure 7C), GFAP (Figure 7F), and TNF-α (Figure 7I) were inhibited by i.t. Ad-PTEN. Quantification of OX-42 (Figure 7J), GFAP (Figure 7K), and TNF-α (Figure 7L) immunoreactivity supported the finding that inhibition of CCI-induced upregulation of OX-42 and GFAP, which are microglial and astrocytic immunohistochemical activation markers, as well as TNF-α, are consistent with the anti-nociceptive effects of i.t. Ad-PTEN.

### The effects of Ad-PTEN on spinal astrocytic PTEN signaling in CCI rats

**Figure 8:**
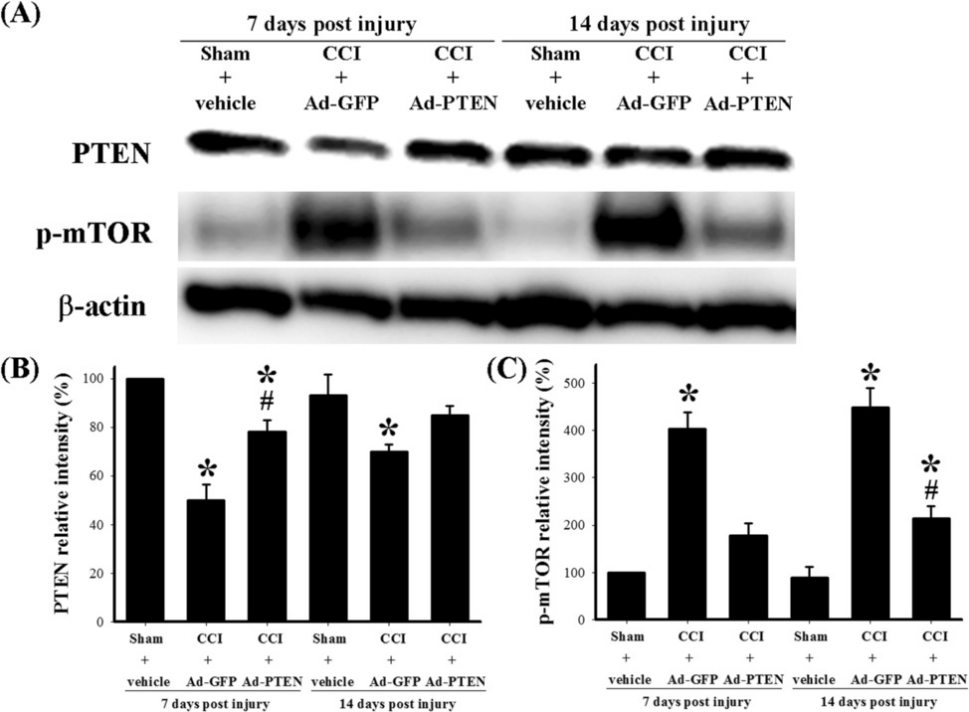
**Effects of the time course of i.t. Ad-PTEN on the spinal PTEN and phospho-mTOR levels in CCI rats. (A)** Western blots for PTEN, phospho-mTOR, and β-actin proteins from sham-operated plus i.t. vehicle 7 days post injury, CCI plus i.t. Ad-GFP 7 days post injury, CCI plus i.t. Ad-PTEN 7 days post injury, sham-operated plus i.t. vehicle 14 days post injury, CCI plus i.t. Ad-GFP 14 days post injury, and CCI plus i.t. Ad-PTEN 14 days post injury. **(B)** Relative density of the immunoblot of PTEN. **(C)** Relative density of the immunoblot of phospho-mTOR. Relative band intensities of PTEN and phospho-mTOR were quantified by densitometry and indicated as the percent change relative to that for the sham-operated 7 days post injury group (100%). Western blotting revealed that PTEN and phospho-mTOR protein expression were detectable in the sham-operated group, but CCI-induced downregulation of PTEN and upregulation of phospho-mTOR protein on day 7 and day 14 after CCI surgery were both attenuated by i.t. Ad-PTEN. Western blotting of β-actin was performed to verify that equivalent amounts of protein were loaded in each lane. Each bar in (B) and (C) represents the mean ± SEM with three rats per group. *P < 0.05 compared with sham-operated 7 days post injury group. ^#^ *P* < 0.05 between CCI plus i.t. Ad-PTEN group compared with the same time points in CCI plus i.t. Ad-GFP group. Ad-GFP, adenovirus-mediated green fluorescent protein; Ad-PTEN, adenovirus-mediated phosphatase and tensin homolog deleted from chromosome 10; CCI, chronic constriction injury; p-mTOR, phosphorylated mammalian target of rapamycin.

**Figure 9:**
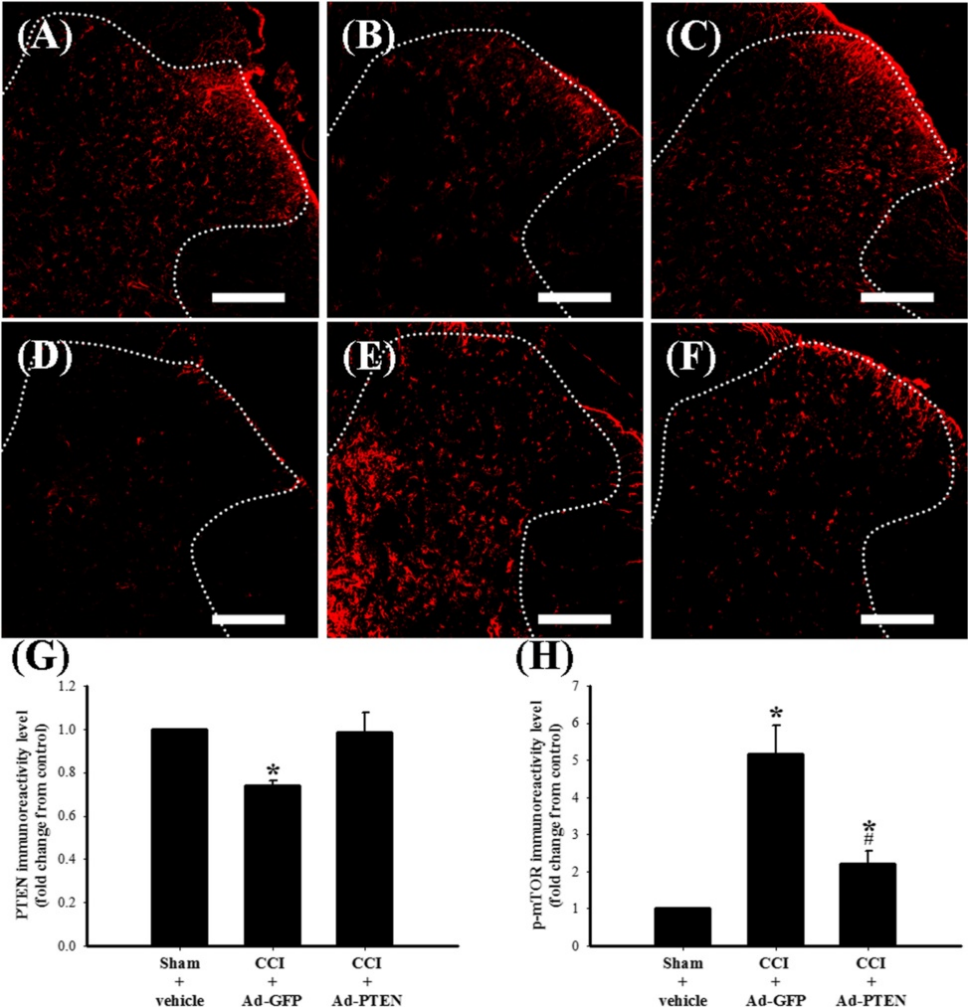
**The inhibitive effect of i.t. Ad-PTEN on CCI-induced downregulation of PTEN and upregulation of phospho-mTOR.** Spinal cord sections (10 μm) obtained 14 days post-surgery from sham-operated plus i.t. vehicle **(A, D)**, CCI plus i.t. Ad-GFP **(B, E)**, and CCI plus i.t. Ad-PTEN **(C, F)** groups. Immunostaining images show cells labeled with PTEN (red) (A-C) and phospho-mTOR (red) (D-F) in the spinal cord. Quantification of PTEN **(G)** and phospho-mTOR **(H)** immunoreactivity in the ipsilateral dorsal horn of the lumbar spinal gray matter, and each bar in (G-H) represents the mean ± SEM with six rats per group. Ad-PTEN (i.t.) significantly attenuated CCI-induced downregulation of spinal PTEN immunoreactivity and upregulation of spinal phospho-mTOR immunoreactivity. Scale bars: 200 μm for all images (A–F). *P < 0.05 compared with sham-operated plus i.t. vehicle group; ^#^ *P* < 0.05 compared with CCI plus i.t. Ad-GFP. Ad-GFP, adenovirus-mediated green fluorescent protein; Ad-PTEN, adenovirus-mediated phosphatase and tensin homolog deleted from chromosome 10; CCI, chronic constriction injury; p-mTOR, phosphorylated mammalian target of rapamycin.

**Figure 10:**
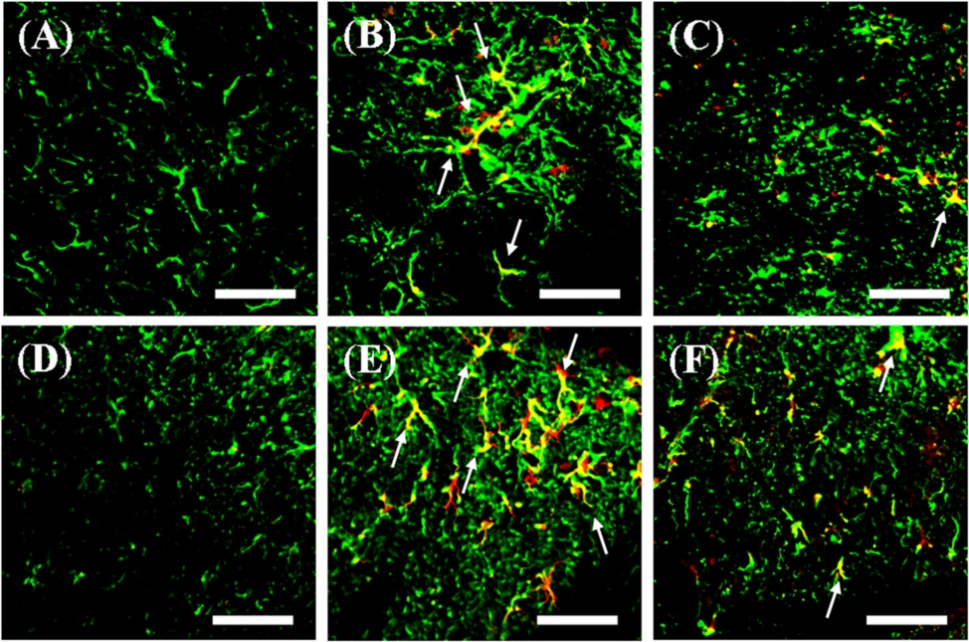
**The effects of i.t. Ad-PTEN on CCI-induced upregulation of TNF-**α **and phospho-mTOR in spinal astrocytes.** Spinal cord sections (10 μm) obtained 14 days post-surgery from sham-operated plus i.t. vehicle **(A, D)**, CCI plus i.t. Ad-GFP **(B, E)**, and CCI plus i.t. Ad-PTEN **(C, F)** groups. Confocal double-immunofluorescent staining of TNF-α (red; A-C) or phospho-mTOR (red; D-F) with GFAP (astrocyte specific marker; green) in the dorsal horn region of the lumbar spinal cord. The merged images (yellow; white arrow) indicate co-localization of TNF-α or phospho-mTOR with GFAP immunoreactive cells in the spinal cord. The confocal results showed that spinal TNF-α and phospho-mTOR were primarily co-localized with astrocytes in the CCI group, whereas i.t. Ad-PTEN significantly attenuated CCI-induced upregulation of spinal TNF-α and phospho-mTOR immunoreactivity in astrocytes. Scale bars are 50 μm for all images.

We further examined the effects of i.t. Ad-PTEN on CCI-induced changes in the spinal PTEN pathway. We further used Western blot analysis to measure changes in PTEN and phospho-mTOR in the dorsal horn of the lumbar spinal cord from the following six groups of rats: (1) sham-operated plus i.t. vehicle 7 days post injury, (2) CCI plus i.t. Ad-GFP 7 days post injury, (3) CCI plus i.t. Ad-PTEN 7 days post injury, (4) sham-operated plus i.t. vehicle 14 days post injury, (5) CCI plus i.t. Ad-GFP 14 days post injury, and (6) CCI plus i.t. Ad-PTEN 14 days post injury. The Western blot analysis revealed both a downregulation of PTEN (Figure 8B) and an upregulation of phospho-mTOR (Figure 8C) in the spinal cord on days 7 and 14 after CCI surgery, which were both attenuated by i.t. Ad-PTEN. Compared with the sham-operated plus i.t. vehicle group (Figure 9A), PTEN immunoreactivity in the CCI plus i.t. Ad-GFP group (Figure 9B) decreased 14 days post injury. Compared with the i.t. Ad-GFP group, i.t. Ad-PTEN (Figure 9C) attenuated CCI-induced downregulation of PTEN 14 days post injury. Compared with the sham-operated plus i.t. vehicle group (Figure 9D), phospho-mTOR immunoreactivity in the CCI plus i.t. Ad-GFP group (Figure 9E) increased 14 days post injury. Compared with i.t. Ad-GFP group, i.t. Ad-PTEN (Figure 9F) attenuated CCI-induced upregulation of phospho-mTOR 14 days post injury. Quantification of the immunoreactivity results further confirmed that i.t. Ad-PTEN significantly attenuated CCI-induced downregulation of PTEN (Figure 9G) and upregulation of phospho-mTOR (Figure 9H) 14 days post injury. To examine the inhibitory effects of i.t. injection of Ad-PTEN on CCI-induced upregulation of TNF-α and phospho-mTOR in spinal astrocytes, we prepared tissue 14 days post-surgery from sham-operated plus i.t. vehicle (Figure 10A,D), CCI plus i.t. Ad-GFP (Figure 10B,E), and CCI plus i.t. Ad-PTEN (Figure 10C,F) rats to perform double-immunofluorescent staining. The confocal double-immunostaining results showed that spinal TNF-α (red; Figure 10B) and phospho-mTOR (red; Figure 10E) were primarily co-localized with astrocytes in the CCI group, and i.t. Ad-PTEN significantly attenuated CCI-induced upregulation of spinal TNF-α (Figure 10C) and phospho-mTOR (Figure 10F) immunoreactivity in astrocytes.

## Discussion

### The role of endogenous PTEN in neuropathic and normal rats

Previous neuroscience studies have focused on the role of PTEN in axon regeneration [11,51,52], Alzheimer’s disease [53], Parkinson’s disease [54], ischemic brain injury [12], and spinal cord injury [52,55-57]. To the best of our knowledge, the present report is the first to explore the role of spinal PTEN in pain, especially neuropathic pain. Using a spinal immunohistofluorescence assay system and Western blot analysis, we found that CCI could elicit downregulation of ipsilateral dorsal spinal PTEN in rats (Figures 1 and 2). Phosphorylation of Ser-380 is known to suppress phosphatase activity of PTEN [58]. We also demonstrated that upregulation of phospho-PTEN was associated with downregulation of PTEN 14 days after CCI surgery (Figures 1 and 2). Phosphorylation of Ser-2448 mTOR is a biomarker for the activation status of mTOR [59], a downstream inhibitory target of PTEN [10-12]. However, there is a lack of information regarding the changes in spinal phospho-mTOR in CCI rats. Indeed, we found that upregulation of phospho-mTOR (Figure 1L) was associated with upregulation of phospho-PTEN (Figure 1J) after CCI surgery. After examining the changes in endogenous PTEN levels in neuropathic rats, we explored the potential effects of modulating spinal PTEN pathway on neuropathic pain. We observed that rats exhibited mechanical allodynia after i.t. injection of Ad-antisense PTEN (Figure 3B), which confirms that downregulation of spinal PTEN is important for nociception, and therefore, we suspect that spinal PTEN exerts beneficial effects against pain.

### The analgesic effects of spinal PTEN

Clinical neuropathic pain syndromes are characterized by evoked pain (hyperalgesia and allodynia) or spontaneous pain [60]. We have confirmed these nociceptive behaviors in the rat CCI model, including thermal hyperalgesia (Figure 5A), mechanical and cold allodynia (Figure 5B,C), and weight-bearing deficits (spontaneous pain; Figure 5D). Ad-PTEN administered i.t. significantly prevented CCI-induced development of pain behaviors in neuropathic rats, including thermal hyperalgesia, mechanical allodynia, and cold allodynia accompanied with weight-bearing deficits (Figure 5). Central sensitization within the dorsal horn of the spinal cord could contribute to the hypersensitive pain behaviors commonly observed with neuropathic pain [61]. Activation of microglia and astrocytes could dominate spinal neuroinflammation [62,63] and accelerate central sensitization as well as the subsequent development and maintenance of neuropathic pain [20,21]. Upregulation of OX-42 (microglial marker) and GFAP (astrocytic marker) immunoreactivity in the spinal dorsal horn are known indicators of elevated nociceptive states [23-25,64-69], which was further confirmed in the CCI rat model by our spinal immunohistofluorescence data (Figure 7). Many studies have demonstrated that inhibition of microglial and astrocytic activation exerts analgesic effects [19,23-25]. Although minocycline, a microglial inhibitor, can prevent neuropathic pain, it cannot reverse established neuropathic pain, thereby showing the important role of spinal microglia in the development phase but not in the maintenance phase of neuropathic pain [25,70,71]. However, it is still controversial whether spinal microglia contributes to the maintenance of nociception in neuropathy. As previously reported by Stuesse *et al*., OX-42 activity substantially peaked at around day 7 after nerve injury, and this increase was maintained for 35 days post-injury [68]. Several studies also suggest that microglial activation is probably important for the maintenance as well as the early development of neuropathic pain [31,72,73]. A series of studies have shown that astrocytic activation is implicated in the maintenance of neuropathic pain [64-67,69], indicating the key role of astrocytes in maintaining neuropathic pain [74,75]. Activated astrocytes are also reported to be important contributors of neuropathic pain development [76]. Our previous studies [19,42,77] and our present findings also indicate that nociceptive behaviors accompany spinal microglial and astrocytic activation (Figure 7) in CCI rats. Moreover, our present findings also demonstrate that the upregulation of spinal PTEN by i.t. Ad-PTEN can significantly attenuate CCI-induced microglial and astrocytic activation in neuropathic rats (Figure 7).

### The majority of spinal cells affected by PTEN

**Figure 11:**
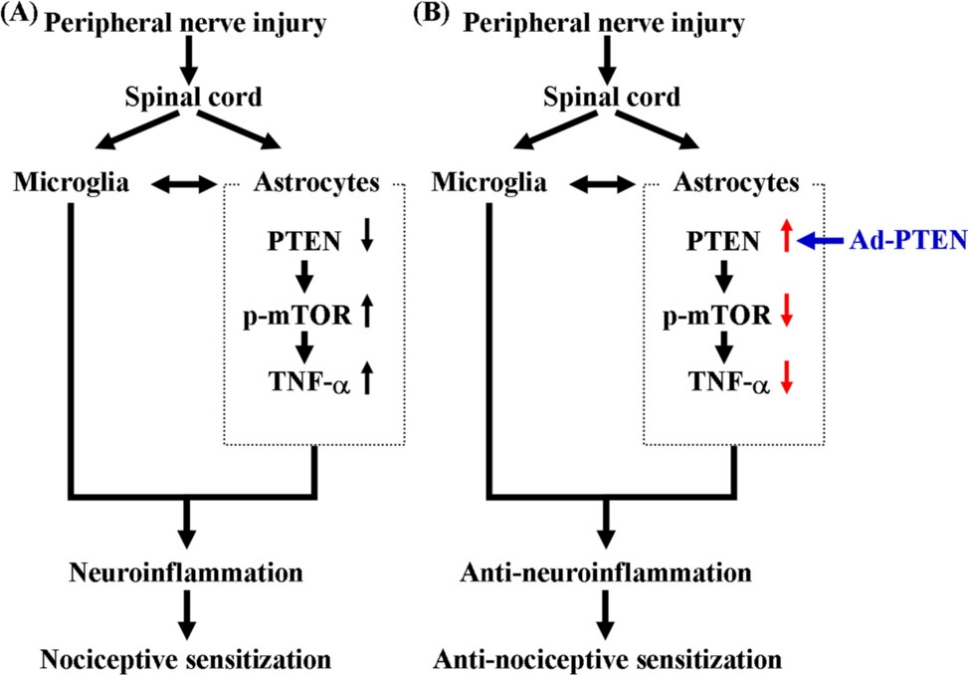
**Schematic representation of the possible mechanisms of PTEN involvement in neuropathic pain. (A)** Peripheral nerve injury could downregulate the spinal astrocytic PTEN, which leads to the upregulation of phospho-mTOR and TNF-α and results in neuroinflammation and nociceptive sensitization. In addition, spinal microglia-astrocyte interactions also promote nociceptive responses. **(B)** Through the upregulation of spinal astrocytic PTEN by i.t. Ad-PTEN, peripheral nerve injury-induced upregulation of phospho-mTOR and TNF-α is inhibited, thereby inhibiting neuroinflammation and nociceptive sensitization. The effect of the upregulation of spinal PTEN by i.t. Ad-PTEN on attenuating peripheral nerve injury-induced microglial activation may be through suppressing the astrocyte-microglia interaction by reducing the astrocytic mediator TNF-α. Ad-PTEN, adenovirus-mediated phosphatase and tensin homolog deleted from chromosome 10; p-mTOR, phosphorylated mammalian target of rapamycin; TNF, tumor necrosis factor.

The expression and localization of PTEN in pain-related regions of the central nervous system are not completely understood. Our confocal double-immunostaining images of the lumbar spinal dorsal gray matter further confirmed that astrocytes are a major source of PTEN expression as compared to neurons and microglia in sham-operated rats (Figure 2). But certainly, the possibility that neurons and microglia could also be the source of PTEN expression cannot be excluded. This finding supports the hypothesis that the spinal astrocytic response plays a crucial role in neuropathic pain [19]. Ad-PTEN administered via i.t. injection significantly upregulated spinal PTEN immunoreactivity (Figure 4D). Previous studies have shown that the majority of spinal cells with adenovirus-mediated target gene overexpression were astrocytes [78,79]. Similar to the above studies, the present confocal results showed that spinal PTEN was primarily co-localized with astrocytes in the i.t. Ad-PTEN group (Figure 4E,F,G). Moreover, our present findings also showed that i.t. Ad-PTEN significantly attenuated CCI-induced astrocytic activation in neuropathic rats (Figure 7). This phenomenon was partially echoed by the finding that PTEN loss resulted in hypertrophy and increased proliferation of astrocytes *in vivo* [80], which are two characteristics of activated astrocytes [81]. Based on the above reports and the present results, we suggest that the prevention of neuropathic pain by i.t. Ad-PTEN may be mainly associated with its inhibitory effects on activated astrocytes, which play key roles in nociceptive hypersensitization. Previous studies have demonstrated that microglia could interact with astrocytes, causing downstream biochemical and behavioral responses to noxious stimuli [82-84]. Additionally, mediators released by activated microglia induce spinal astrocyte activation, and blocking these mediators attenuates astrocyte activation and pain [82-85]. Furthermore, mediators released by activated astrocytes, in turn, also activate spinal microglia in neuropathic or focal brain-injured rodents [86,87]. These findings from previous studies suggest that spinal microglia-astrocyte interactions could promote nociceptive responses (Figure 11A). Therefore, in this study, we hypothesized that the effect of the upregulation of spinal PTEN by i.t. Ad-PTEN on attenuating CCI-induced microglial activation may be secondary to the direct inhibition of activated astrocytes through suppressing the astrocyte-microglia interaction by reducing the release of astrocytic mediators (Figure 11B).

### Potential analgesic mechanisms of PTEN

Previous studies have found that the activation of spinal mTOR is required for CCI-induced pain hypersensitivity [47]. Inhibition of the spinal mTOR pathway by i.t. rapamycin [13] or spinally administered rapamycin ester analogue CCI-779 [14] attenuates mechanical hyperalgesia in neuropathic rats. The above studies suggested that the anti-nociceptive effects produced by inhibition of the spinal mTOR pathway in neuropathic rats are consistent with a deleterious role for mTOR in neuropathic rats. In the present study, we observed upregulation of phospho-mTOR following CCI surgery (Figure 1L). The findings of cancer research studies have indicated that PTEN is an upstream inhibitory mediator of mTOR, which also has been confirmed in the central nervous system (CNS) [10-12]. Our results indicated that i.t. Ad-PTEN significantly attenuated CCI-induced upregulation of spinal phospho-mTOR immunoreactivity (Figures 8 and 9). Moreover, similar to the systemic anti-inflammatory effects of intra-articular Ad-PTEN [17], i.t. Ad-PTEN also has central anti-inflammatory effects and can inhibit CCI-induced upregulation of spinal TNF-α (Figure 7). TNF-α, a neuropathic pain-related cytokine [26], caused thermal hyperalgesia and mechanical allodynia in rats [27]. Moreover, neuropathic mechanical allodynia in rats was blocked by i.t. injection of a TNF-soluble receptor, a TNF antagonist [28]. In the present study, the confocal results from CCI rats showed that upregulated phospho-mTOR and TNF-α were localized predominantly to astrocytes, and this upregulation was inhibited by i.t. Ad-PTEN (Figure 10). Rapamycin, a specific inhibitor of mTOR, was reported to inhibit TNF-α release from human vascular smooth muscle cells *in vitro* [88]. Other studies have reported that rapamycin inhibition of mTOR in microglia [89] and astrocytes [90] produces anti-inflammatory effects, which suggests that inhibiting mTOR can reduce neuroinflammation [91]. Thus, according to the previous and present findings, we conclude that one analgesic mechanism of PTEN may be inhibition of the astrocytic inflammatory response (upregulated TNF-α) via inhibition of phospho-mTOR (Figure 11B).

### Advantages and future studies of PTEN for neuropathic pain

We suspect that the upregulation of PTEN may be therapeutic for neuropathic pain, a neuroinflammation-related disorder. Our present results demonstrate that PTEN exerts beneficial effects against neuropathic pain, which highlights three future research directions. First, it is important and valuable to explore the functions of PTEN through neuroscience research, especially neuroinflammation-related disorder mediated by activated astrocytes. However, a few *in vitro* [89] and *in vivo* [92] studies showed that inhibition of mTOR may modulate neurons or microglial activation. Therefore, the possibility that i.t. Ad-PTEN directly affects spinal neurons or microglia cannot be excluded. Second, Sims *et al*. [93] considered the adenovirus to be an efficient and safe vector for CNS gene delivery, although there are limitations to adenoviral-mediated PTEN gene therapy. The individuals treated with adenoviral vectors were previously reported to have experienced systemic reactions including fever, chill, and hypertension [48]. Although i.t. Ad-PTEN did not significantly affect the response to thermal hyperalgesia, mechanical allodynia, or cold allodynia tests on the contralateral hindpaw in CCI rats (Figure 6). Additionally, i.t. Ad-PTEN-treated normal rats and CCI rats did not exhibit any obvious abnormal external behavior (including locomotor function). The minimal effective dose and the duration of PTEN gene therapy still need to be determined in future research. Third, there is no known PTEN activator for neuropathic pain, and upregulation of PTEN may serve as an *in vitro* index to screen compounds that may be potential neuropathic pain therapies.

## Conclusions

This study is a novel step toward understanding the benefits of PTEN on neuropathic pain and provides a foundation for future studies utilizing upregulation of PTEN in combinational therapies for neuropathic pain.
